# Author Correction: Commensal production of a broad-spectrum and short-lived antimicrobial peptide polyene eliminates nasal *Staphylococcus aureus*

**DOI:** 10.1038/s41564-024-01798-4

**Published:** 2024-08-08

**Authors:** Benjamin O. Torres Salazar, Taulant Dema, Nadine A. Schilling, Daniela Janek, Jan Bornikoel, Anne Berscheid, Ahmed M. A. Elsherbini, Sophia Krauss, Simon J. Jaag, Michael Lämmerhofer, Min Li, Norah Alqahtani, Malcolm J. Horsburgh, Tilmann Weber, José Manuel Beltrán-Beleña, Heike Brötz-Oesterhelt, Stephanie Grond, Bernhard Krismer, Andreas Peschel

**Affiliations:** 1 Department of Infection Biology, Interfaculty Institute of Microbiology and Infection Medicine, University of Tübingen, Tübingen, Germany; 2 Cluster of Excellence EXC 2124 Controlling Microbes to Fight Infections, Tübingen, Germany; 3 German Center for Infection Research (DZIF), partner site Tübingen, Tübingen, Germany; 4 Present Address: Institute of Organic Chemistry, University of Tübingen, Tübingen, Germany; 5 Department of Microbial Bioactive Compounds, Interfaculty Institute of Microbiology and Infection Medicine, University of Tübingen, Tübingen, Germany; 6 Institute of Pharmaceutical Sciences, University of Tübingen, Tübingen, Germany; 7 Department of Laboratory Medicine, Renji Hospital, School of Medicine, Shanghai Jiaotong University, Shanghai, China; 8 Department of Infection Biology and Microbiomes, University of Liverpool, Liverpool, UK; 9 The Novo Nordisk Foundation Center for Biosustainability, Technical University of Denmark, Kongens Lyngby, Denmark

**Keywords:** Antibiotics, Natural products

Correction to: *Nature Microbiology* 10.1038/s41564-023-01544-2, published online 18 December 2023.

This article was originally published under standard Springer Nature license (© The Author(s), under exclusive licence to Springer Nature Limited). It is now available as an open-access paper under a Creative Commons Attribution 4.0 International license, © The Author(s). The error has been corrected in the HTML and PDF versions of the article.

