# Supplementary material for: Commensal production of a broad-spectrum and short-lived antimicrobial peptide polyene eliminates nasal Staphylococcus aureus
Source: Nat Microbiol. 2023 Dec 18;9(1):200–13. doi: 10.1038/s41564-023-01544-2 (PMC11310079; doi:10.1038/s41564-023-01544-2)
Supplement: Supplementary file 1 — Supplementary Figs. 1–8, information and Tables 1–7. [file 41564_2023_1544_MOESM1_ESM.pdf]

nature microbiology

Supplementary information

https://doi.org/10.1038/s41564-023-01544-2

Commensal production of a broad-spectrum and short-lived antimicrobial peptide polyene eliminates nasal *Staphylococcus aureus*

In the format provided by the authors and unedited

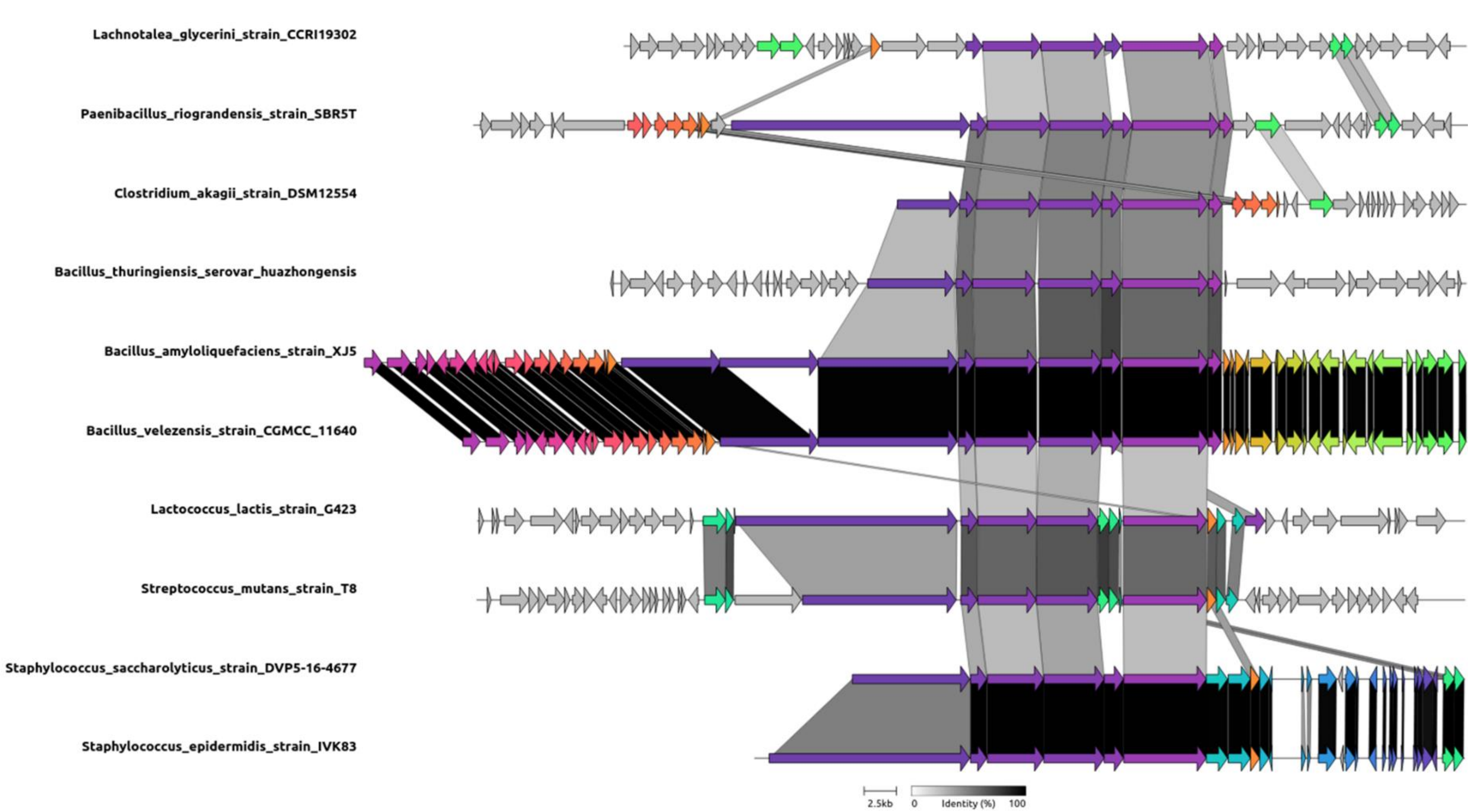

Supplementary Figure 1. Comparison of the genetic organization of the epifadin gene cluster with homologous BGCs from NCBI database. The arrow positions represent gene orientation. Arrow colours indicate clusters of different gene functions as predicted by antiSMASH (v6.1.1). Representative bacterial strains in which the BGCs were identified are indicated on the left. Whereas the epifadin BGC shows extremely high identity in gene organisation and sequence to the *S. saccharolyticus* BGC, all other bacteria exhibit only a high level of similarity at the gene organisation level but limited identity at the nucleotide level. This suggests that the homologous BGCs may not encode for epifadin but similar compounds. Obviously, the BGCs from *B. amyloliquefaciens* and *B. velezensis* are extremely similar to each other, but *B. amyloliquefaciens* has acquired an additional NRPS gene not present in *P. velezensis*.

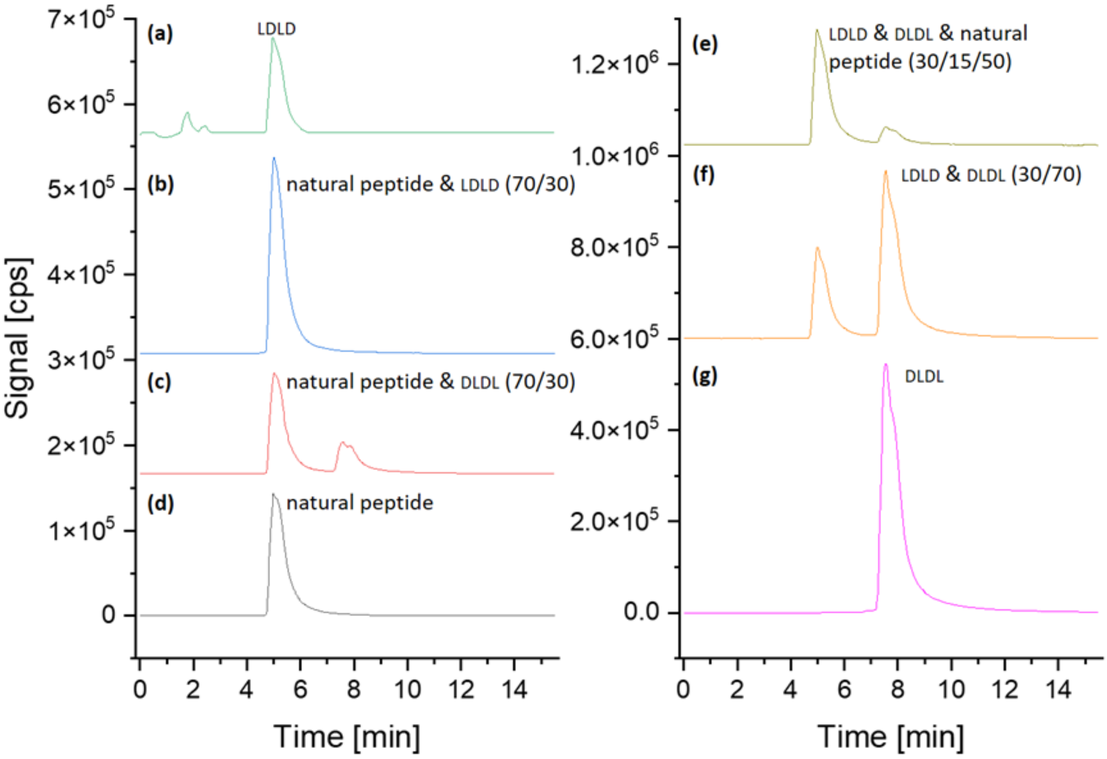

Supplementary Figure 2. Extracted ion chromatograms (EICs, *m/z* = 541.2405 ± 0.05) of the analyses of the peptide amides were recorded with a HPLC-ESI-QTOF-MS. All pure samples had a concentration of 0.1 mg/mL. **(a)** The EIC of the synthetic L-D-L-D peptide (green). **(b)** The EIC (blue) shows a mixture (70/30) of the isolated natural peptide and the synthetic L-D-L-D peptide. **(c)** A mixture (70/30) of the isolated natural peptide and the synthetic D-L-D-L peptide is shown (EIC, red). **(d)** The EIC of the natural peptide purified from culture extract is shown (black). **(e)** The olive EIC shows a ternary mixture (30/15/50) of the synthetic L-D-L-D peptide, synthetic D-L-D-L peptide and the isolated natural peptide. **(f)** The mixture (30/70) of the synthetic L-D-L-D peptide and synthetic D-L-D-L peptide shows the given EIC (orange). **(g)** The EIC of the synthetic D-L-D-L peptide is presented (purple).

(A)

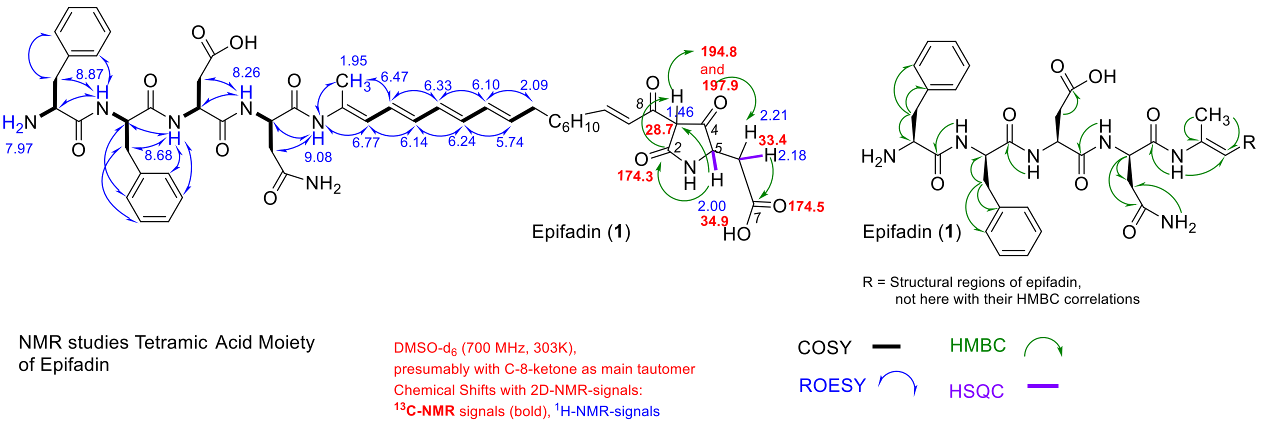
Epifadin (1)
Epifadin (1)
R = Structural regions of epifadin, not here with their HMBC correlations
NMR studies Tetramic Acid Moiety of Epifadin
DMSO-d6 (700 MHz, 303K), presumably with C-8-ketone as main tautomer
Chemical Shifts with 2D-NMR-signals: 13C-NMR signals (bold), 1H-NMR-signals
COSY
HMBC
ROESY
HSQC

(B)

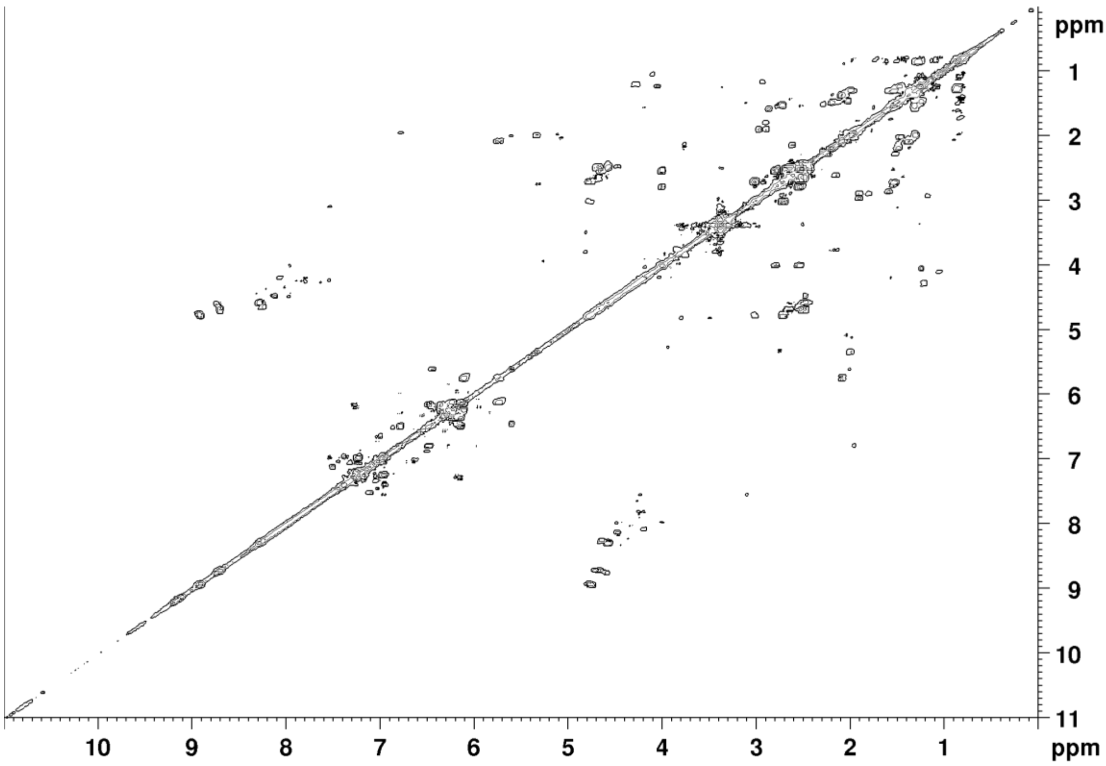
ppm
ppm

(C)

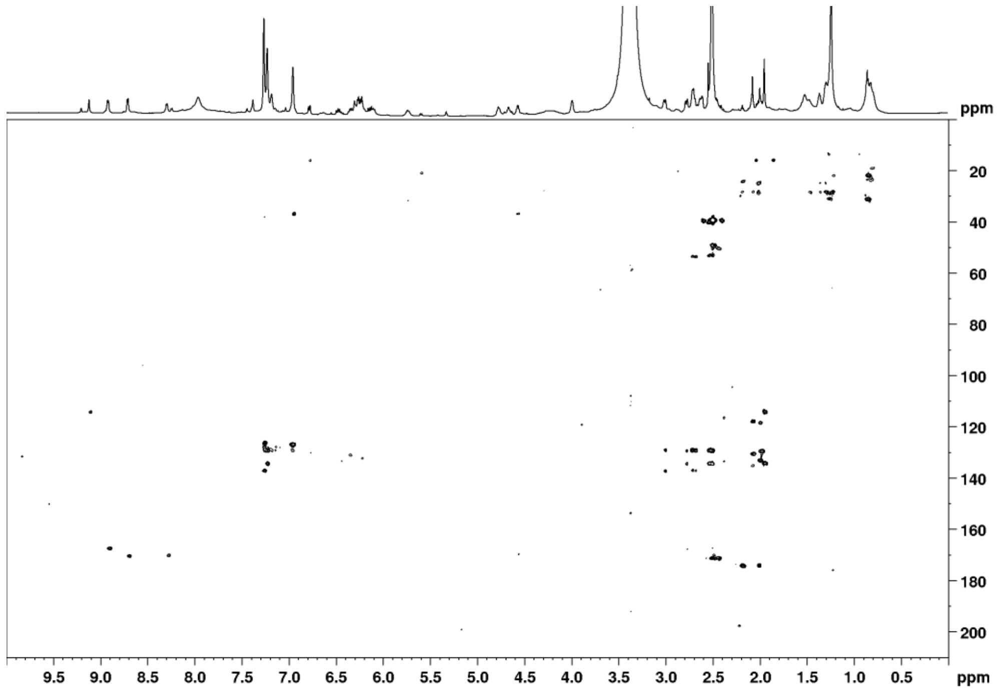

(D)

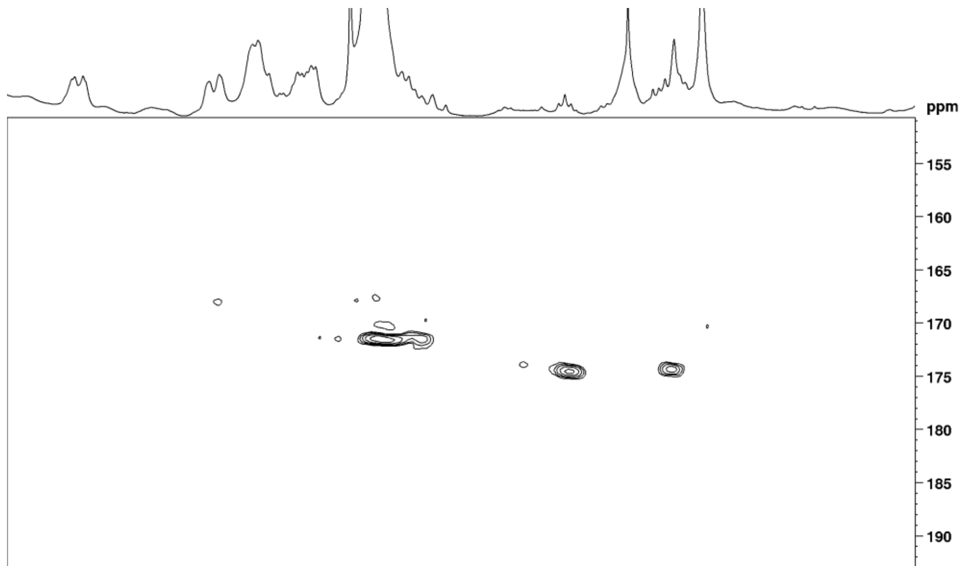

Supplementary Figure 3. Correlations of 2D-NMR experiments of epifadin (1) with focus on its tetramic acid. (A) In the structure of epifadin (**1**), arrows in blue show ROESY correlations, arrows in green show HMBC correlations, bold black bonds show COSY correlations and bold purple bonds show HSQC correlations. ^{1}H- (blue) and ^{13}C-NMR (red) shifts are given in ppm. (**B**) ^{1}H-^{1}H-COSY spectrum of epifadin (**1**) in DMSO-d_6 (700 MHz, 303 K). **(C)** ^{1}H-^{13}C-HMBC spectrum of epifadin (1) in DMSO-d_6 at 303 K (700 MHz). **(D)** Expansion of ^{1}H-^{13}C-HMBC spectrum of epifadin (**1**) in DMSO-d_6 at 303 K (700 MHz). Key correlation of tetramic acid moiety tautomer at 197.9 ppm (^{13}C) and 2.21 ppm (^{1}H). The tetramic acid moiety of epifadin (**1**) is assigned with its three carbonyl groups from the NMR-experiments in dmso-d_6.

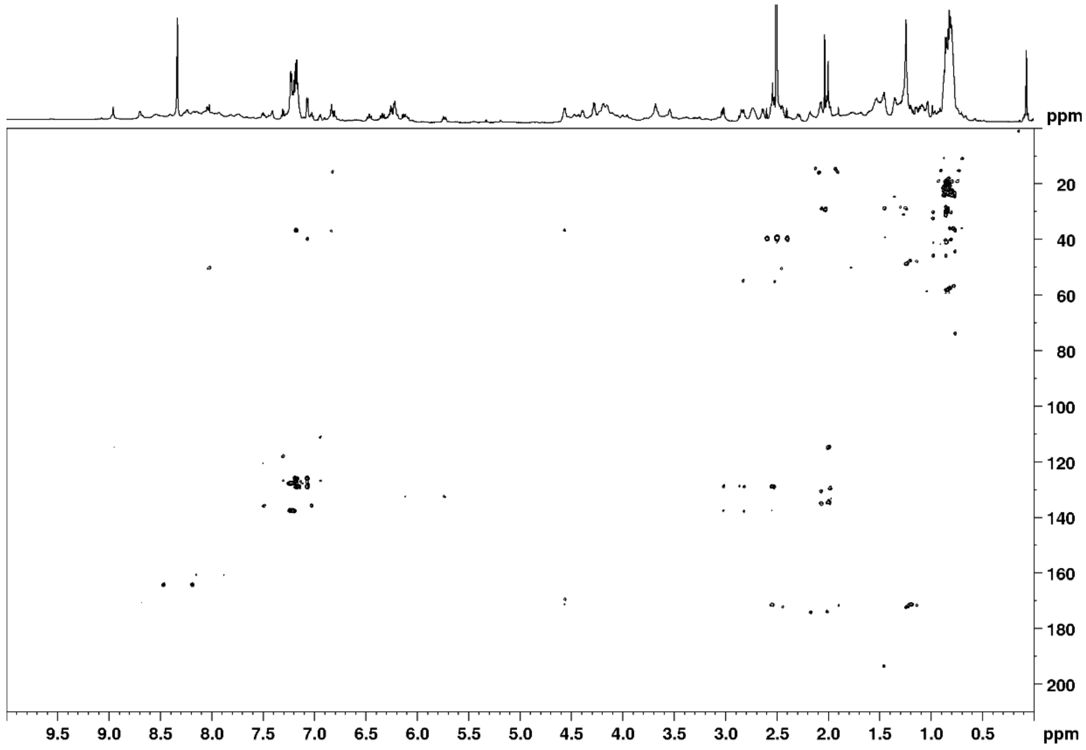

(A)

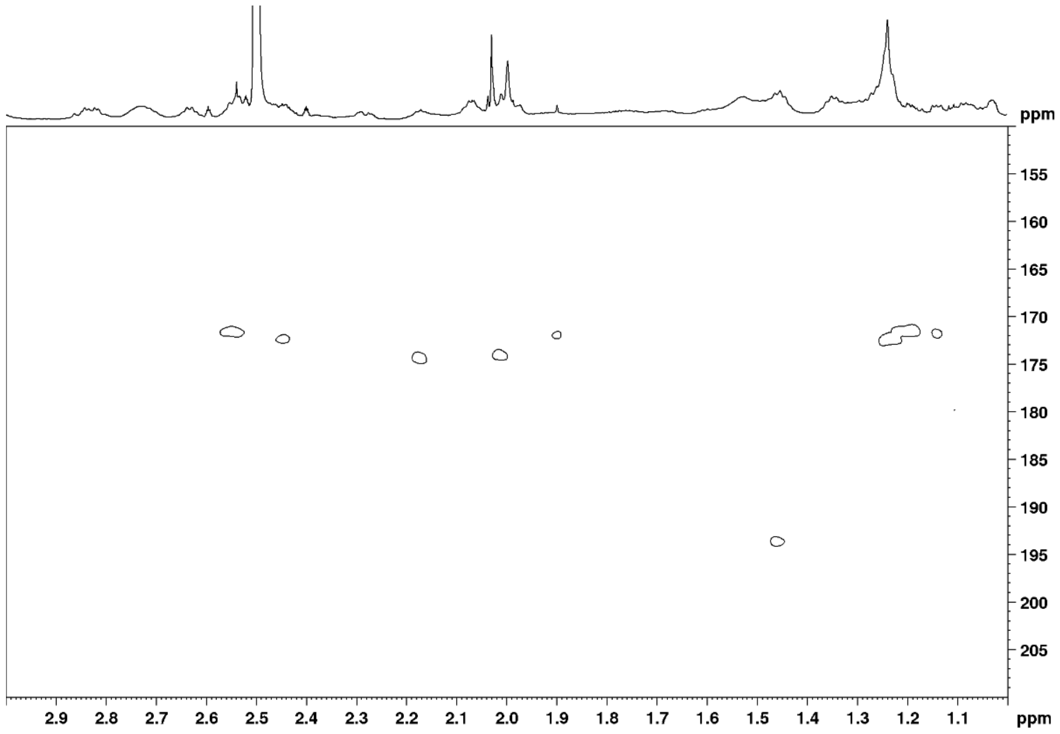

(B)

Supplementary Figure 4. 2D-NMR of epifadin (1) at 308 K. (A) ^{1}H-^{13}C-HMBC spectrum of epifadin (**1**), different batch, DMSO-d_6, 308 K, 700 Mhz. Here, this tetramic acid tautomer shows the correlation signal with the 194.8-^{13}C-NMR signal. **(B)** Expansion of ^{1}H-^{13}C-HMBC spectrum of epifadin (**1**), different batch, in DMSO-d_6 at 308 K. Key correlation of tetramic acid moiety at 194.8 ppm (^{13}C) and 1.46 ppm (^{1}H).

47

1. Methylation reaction experiments towards methylated epifadin (1) with its tetramic acid moiety.

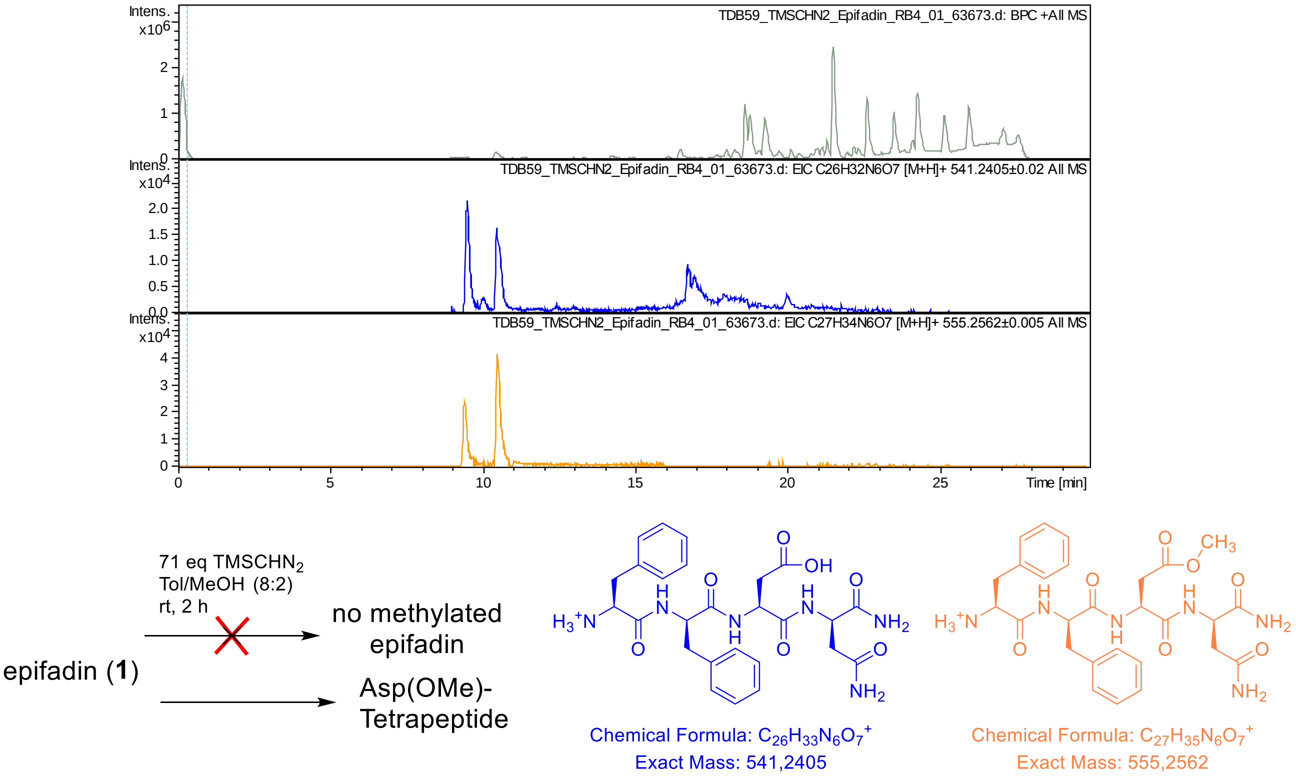

Supplementary Figure 5. HPLC-HRMS analysis of the methylation reaction products of epifadin (1) with trimethylsilyldiazomethane. Chemical methylation of epifadin yielded only the methylated peptide and no methylated full epifadin analogue. Any polyene-tetramic acid fragments or analogues thereof were not detected. Base peak chromatogram of the reaction mixture is shown in grey, EIC of the peptide amide **2** is shown in blue and EIC of the methylated peptide amide **2** is shown in orange. Structures of the quasimolecular ion of the peptide amide **2** is shown in blue and of the methylated peptide amide **2** is shown in orange.

2. Methylation reaction experiments towards methylated reutericyclin (6) with its tetramic acid moiety.

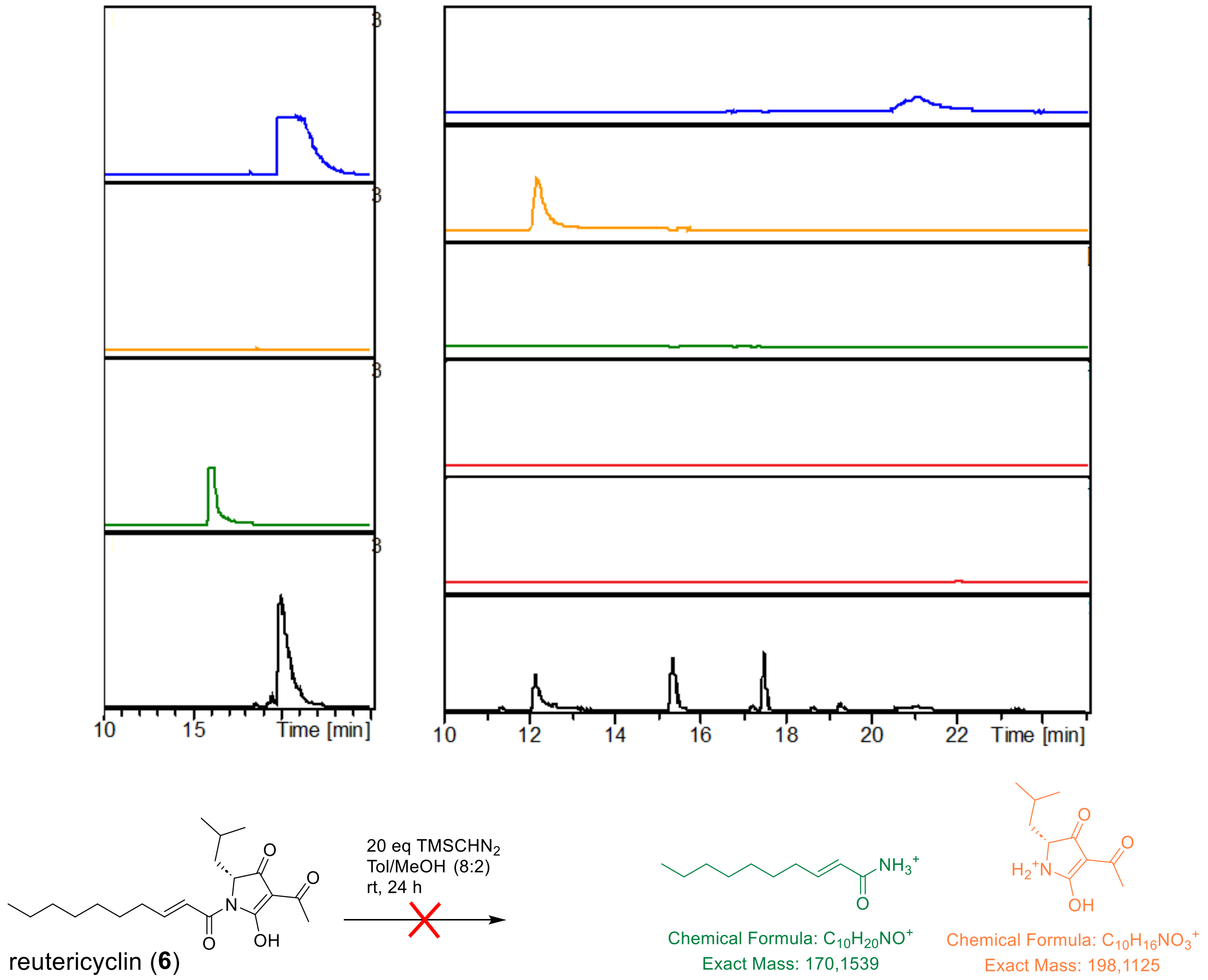

Supplementary Figure 6. HPLC-HRMS analysis of the methylation reaction of reutericyclin (6) with trimethylsilyldiazomethane. EIC of reutericyclin (**6**) is shown in blue, EIC of the tetramic acid head (decomposition) is shown in orange, EIC of the amide chain of reutericyclin (impurity) is shown in green, EICs of mono methylated and dimethylated reutericyclin is shown in red, and UV-chromatogram (280 nm) is shown in black. Left, reaction at t = 0 h. Right, reaction at t = 24 h. Two new unknown peaks can be observed in the UV-chromatogram at 15.3 min and 17.5 min, which cannot be assigned to any methylation or degradation product. Quasimolecular ions of the tetramic acid head (decomposition, orange) and the amide chain of reutericyclin (impurity, green) are shown.

3. Methylation reaction to methylated pyridone bearing kirromycins.

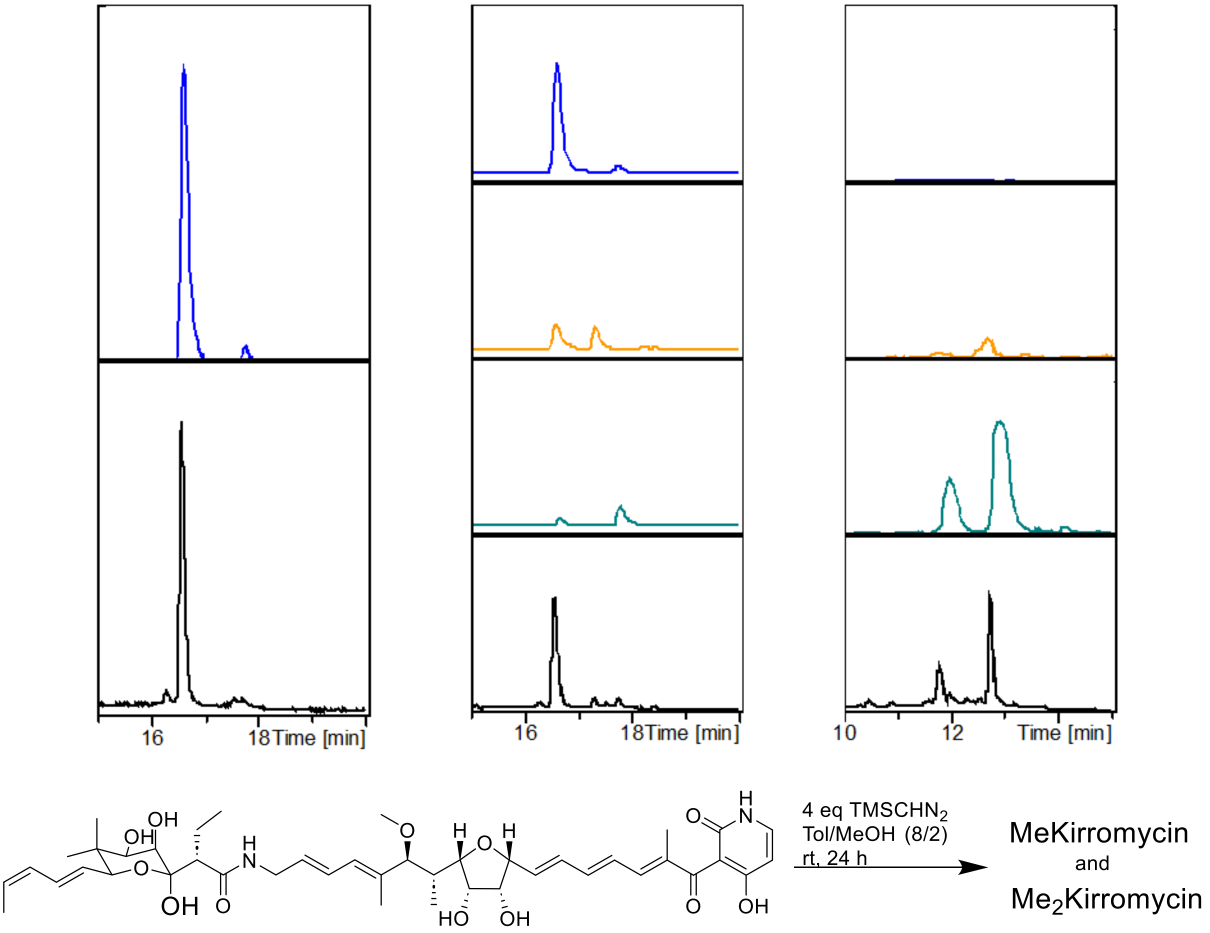

kirromycin (**7**)

Supplementary Figure 7. HPLC-HRMS analysis of the methylation reaction of kirromycin (6) with trimethylsilyldiazomethane. EIC of kirromycin (**7**) is shown in blue, EIC of mono methylated kirromycin is shown in orange, EIC of di-methylated kirromycin is shown in green and UV-chromatogram (350 nm) is shown in black. Left, reaction at t = 0 h. Center, reaction at t = 4 h. Right, reaction at t = 24 h after adding an additional 8 eq trimethylsilyldiazomethane.

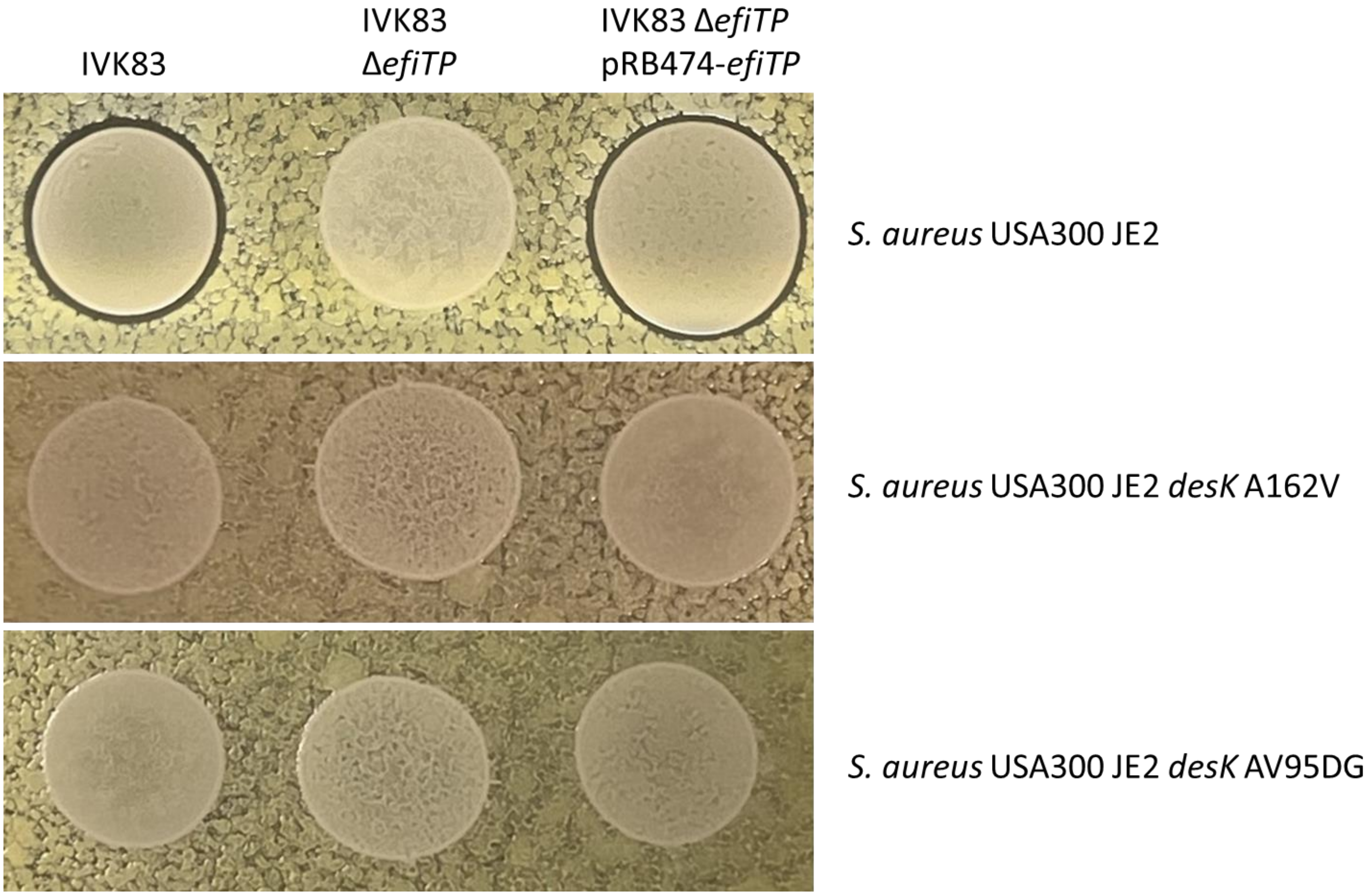

Supplementary Figure 8. Role of *S. aureus desK* genotype in *S. epidermidis* IVK83 growth inhibition. Central spots of *S. epidermidis* strains (IVK83, IVK83 Δ*efiTP* and IVK83 Δ*efiTP* pRB474-*efiTP*) were cultured simultaneously on agar plates spread with either *S. aureus* wild type or *desK* variant strains. Representative image from triplicate independent experiments.

Supplementary Information

Analysis of the epifadin BGC with antiSMASH 5.0[28] predicted a three-partite composition with an *N*-terminal NRP part followed by a PK moiety and a C-terminal single amino acid residue (Fig. 1c).

The NRPS1 (EfiA) enzyme was predicted to start the biosynthesis with an aromatic amino acid. Since the first adenylation domain (A-domain) is followed by two condensation domains (C-domain) the first amino acid was suggested to be incorporated twice, with the second to be converted to D-configuration by the second C-domain with its epimerization domain (Fig. 1c). The second A-domain of EfiA was predicted to incorporate aspartate, the third asparagine, which is predicted again to be epimerized to D-asparagine. The following amino acid position did not yield a clear prediction. According to antiSMASH it could be a small or an aliphatic amino acid such as glycine, alanine, valine, leucine, isoleucine, or amino-butyric acid.

After stabilizing pure epifadin in DMSO-PA (palmitoyl ascorbate) solution, samples were applied immediately to antimicrobial activity assays. Nevertheless, chemical analysis by coupled HPLC-UV/VIS-MS of the intact compound revealed a continuous degradation of epifadin after prolonged incubation at room temperature within hours with the N-terminal peptide-amide as main degradation product. Storage at -80°C tremendously improved the stability of epifadin in DMSO-PA. Tandem MS (MS/MS) experiments of intact epifadin yielded specific fragmentation patterns that supported the predicted peptide sequence (Extended Data Fig. 6). The determined structure of the peptide amide was further confirmed by acetylation and esterification reactions with the natural product extract (Extended Data Fig. 9). According to antiSMASH and the module organization of EfiA, the peptide amide should have an L-D-L-D-amino acid configuration (Fig. 1 and Extended Data Fig. 1). L-Phe-D-Phe-L-Asp-D-Asn-NH_2 (FfDn-NH_2) and its enantiomer D-Phe-L-Phe-D-Asp-L-Asn-NH_2 (fFdN-NH_2) were synthesized by chemical solid-phase synthesis, employing the respective D- or L-asparagine rink-amide resin, and purified. The retention times on a HPLC-RP-C_{18} column as well as the MS/MS spectra of the synthetic molecules were in accordance with the ones of the natural tetra peptide

amide (Extended Data Fig. 1). In order to unambiguously confirm the absolute stereo configuration of the peptide moiety, the natural peptide amide was isolated from a decomposed epifadin NMR sample and both synthetic peptides and the natural peptide were analyzed by HPLC coupled MS using a column with a zwitterionic chiral stationary phase (CHIRALPAK® ZWIX(+)). By comparing the retention times the L-Phe-D-Phe-L-Asp-D-Asn-NH_2 was confirmed as the correct stereo configuration (Supplementary Fig. 2).Towards structure elucidation, a combination of ^{1}H-NMR and 2D-NMR experiments (^{1}H-^{1}H-ROESY, ^{1}H-^{1}H-COSY, and ^{1}H-^{13}C-HMBC) fully assigned and confirmed the presence of four unmodified amino acids, the N-terminal L-Phe, followed by D-Phe, L-Asp, and D-Asn, consistent with the sequence deduced from MS/MS (Extended Data Figs. 3,4a). Additionally, NMR revealed the fifth amino acid to be a modified L-alanine (Extended Data Fig. 3). This adjacent modified L-Ala residue lacks the C-terminal carbonyl group (C=O) as indicated by the NMR signals for the corresponding enamide moiety to resemble the -NH-C(CH_3)=CH-structure. ^{1}H-^{1}H correlation spectra (Extended Data Fig. 3 and 4) support a tetraene moiety attached to the peptide moiety and indicate an *all-trans* configuration.

Furthermore, correlations of 2D-NMR spectra support the tetramic acid structure (Supplementary Figures 3 and 4). Typical ^{13}C-NMR signals of the keto and enol groups of tetramic acid are in the range between 170 ppm and 200 ppm (see penicillenol G1 and G2[75], more recently MCA17-1[76]), which can also be found in 2D-NMR spectra of epifadin (**1**). However distinct signals of the tetramic acid moiety were not found. This phenomenon was also observed by another research group, which could not detect distinct signals in tetramic acid compounds such as militarinone C (**3**)[77] or pyranonigrin I (**5**) and J (**4**)[78].

Typical derivatization approaches such as acetylation with acetic anhydride and pyridine or methylation with trimethylsilyldiazomethane in toluene/methanol in order to stabilize epifadin (**1**) has led to its decomposition. However, derivatized peptide amide fragment of **1** was observed via HPLC-HRMS analysis (Supplementary Figure 5). We also tested reutericyclin (**6**) and kirromycin (**7**) in a methylation reaction with trimethylsilyldiazomethane in toluene/methanol to optimize conditions

for the methylation reaction of a purified epifadin (**1**) sample. Interestingly, only the methylation of the pyridine-ring containing kirromycin analogue yielded mono- as well as di-methylated kirromycin analogue (Supplementary Figure 7). The methylation of the tetramic acid reutericyclin (**6**) under the same conditions did not yield any mono- or multiple-methylated reutericyclin analogues (Supplementary Figure 6), which indicates that a tetramic acid might not be prone to this methylation method, which is the same in the case for methylation reaction of epifadin (**1**).

Since the derivatization reactions of epifadin (**1**) only led to its decomposition we used additives in the NMR solvents with the aim to stabilize one tautomer and prevent its decomposition. Additives such as $ZnCl_2$, formic acid, or trifluoroacetic acid yielded only NMR spectra, which could not be analyzed. Changing the solvent to deuterated acetonitrile or methanol, we observed that epifadin (**1**) is poorly soluble in these solvents. Using formic acid or trifluoroacetic acid as additives resulted in quick dissolution of the residue. However, recording NMR spectra of the given solution resulted in decomposition.

The epifadin BGC encodes three trans AT-type PKSs (EfiB, EfiC, EfiD) and a hybrid PKS-NRPS (EfiE). EfiB represents a putative discrete AT/free-standing ACP S-malonyltransferase. AntiSMASH suggested that the PK extender units of the EfiB AT domain are derived from malonyl-CoA (Fig. 1c,d). This assumption is supported by the presence of the GHSxG motif (amino acid position 90-94 in EfiB), which is usually conserved in functional AT domains and also by the substrate-binding motif NAFH (amino acid position 197-200 in EfiB), specific for malonyl-CoA[79]. The modules of EfiC and EfiD are probably responsible for the iterative condensation of the acetate extender units, which remain probably unsaturated because EfiC and EfiD contain ketosynthase (KS), peptidyl carrier protein (P), dehydratase (DH) and ketoreductase (KR) domains, of which the latter two generally catalyze double bond formation in polyketides. The PKS module of EfiF consists only of a KS and an ACP domain. The exact chemical structure of the PK part of epifadin could not be fully elucidated by only analytical methods as MS/MS or NMR spectroscopy, because of the extraordinary instability of epifadin. The naturally occurring and ionization-induced fragments are unsaturated PK moieties, which are difficult

to detect by MS because they are often not prone to ionization. The unusual UV absorption maximum at 383 nm supports the presence of an unsaturated polyene PK moiety (Fig. 3). Similar, albeit not fully identical UV absorption properties have also been found in the macrocyclic PK compound amphotericin B[80], which contains seven conjugated double bonds, in the antifungal PK sugar macrocyclic agent nystatin A1 with four plus two conjugated double bonds, and in militarinone C with four conjugated double bonds representing a polyenoyl tetramic acid[77].

antiSMASH predicted that the adenylation domain A_5 of the NRPS module of EfiE is specific for activating aspartate, which is subsequently linked to the PK part of epifadin. HPLC-coupled high resolution MS (MS/MS) analyses of epifadin indicated the modification of aspartate, to yield a tetramic acid as structural feature of epifadin. MS analyses delivered a characteristic signal pattern for the tetramic acid moiety, containing ions with well-assigned fragment ions (e.g., $[M+H]^+$, $C_6H_8NO_4^+$, calculated m/z 158.0448, found 158.0454, Δ3.9 ppm; $[M+H]^+$, $C_6H_6NO_3^+$, calculated m/z 140.0342, found 140.0348, Δ4.1 ppm) (Extended Data Fig. 5). The proposed, chemical fragmentation mechanisms corresponded to the fragmentation observed by MS/MS, thereby supporting the heterocyclic tetramic acid structure (Extended Data Fig. 5). Unfortunately, the method of NMR gave only an incomplete set of signals, presumably as a result of pronounced tautomeric effects of the distinctly charged tetramic acid tautomers. Nevertheless, the genetic architecture of the BGC suggests that the release of the final molecule from the terminal NRP domain is probably catalyzed by cyclization to form the tetramic acid via the terminal C-domain of EfiE and, presumably, the thioesterase EfiT (Fig. 1). Condensation of the PK part with the aspartate residue and the subsequent Dieckmann cyclization catalyzed by the unusual C-terminal condensation domain of EfiE could form the tetramic acid moiety in analogy to a mechanism postulated for malonomycin biosynthesis[30].

Since five individual *S. epidermidis* isolates with the nearly identical plasmid were isolated from three different geographical locations, frequent horizontal gene transfer (HGT) seems obvious. To identify a putative mechanism of HGT, plasmid sequences were further analyzed for the presence of genes involved in transfer. A putative relaxase gene of the newly described Firmicutes-specific MobL-family

of relaxases was identified next to an origin of transfer (*oriT*) region, which clearly suggests horizontal dissemination by conjugation[81].

Supplementary Table 1: Signals of the proton NMR spectrum of the synthetic peptide amide FfDn-NH_2 (DMSO-d_6, 600MHz, 303K).

#	δ_H (mult., *J*)	δ_C	HMBC (H → #C)
**1**			
**2**	3.97 (m)	53.5	3, 4, 8
**3a, 3b**	2.56 (dd, 8.6, 14.2), 2.81(dd, 4.5, 14.2)	37.1	2, 4, 5, 8
**4**		134.9	
**5**	7.00 (d, 7.4)	129.4	3, 4, 5, 6, 7
**6**	7.23 (m)	128.4	4, 5, 7
**7**	7.23 (m)	127.0	4, 5, 6
**8**		168.3	
**9**	8.86 (d, 8.4)		8, 10, 11
**10**	4.69 (m)	54.0	11, 12, 16
**11a, 11b**	2.72 (dd, 10.0, 13.5), 3.00 (dd, 4.4, 14.2)	38.0	10, 12, 13, 16
**12**		137.3	
**13**	7.25 (m)	129.3	11, 12, 14, 15
**14**	7.26 (m)	128.1	12, 13, 15
**15**	7.18 (m)	126.5	12, 13, 14
**16**		170.8	
**17**	8.71 (d, 7.7)		16, 18, 19
**18**	4.55 (m)	49.6	16, 19, 20, 21
**19a, 19b**	2.62 (dd, 4.8, 16.7), 2.50 (overlapping with solvent)	36.4	18, 20, 21
**20**		171.7	
**21**		170.0	
**22**	8.09 (d, 8.2)		21, 23, 24
**23**	4.47 (m)	49.6	21, 24, 25, 27
**24**	2.48 (overlapping with solvent)	36.8	23, 25, 27
**25**		171.9	
**26**	6.88 (s), 7.36 (s)		24, 25
**27**		172.8	
**28**	7.08 (s), 7.11 (s)		23, 27

The scale shows the chemical shift *δ* in parts per million (ppm).

Supplementary Table 2: Calculated and found *m/z* values of corresponding sum formulas. Error is indicated as Δppm.

Sum formula $[M+H]^+$	Calculated *m/z* value	Found *m/z* value	Δppm
$C_{51}H_{62}N_7O_{12}^+$	964.4451	964.4472	2.2
$C_{26}H_{33}N_6O_7^+$	541.2405	541.2422	3.1
$C_{25}H_{33}N_2O_5^+$	441.2384	441.2396	2.8
$C_{25}H_{30}NO_5^+$	424.2118	424.2131	2.8
$C_6H_8NO_4^+$	158.0448	158.0454	3.9
$C_6H_6NO_3^+$	140.0342	140.0348	4.1

Supplementary Table 3: Inhibitory concentration (IC*) of epifadin against the listed bacterial strains.

Antibiotic	Strain	IC* (µg/ml)	IC* (µM)
Epifadin	*S. aureus* USA300LAC	1.5	1.55
	S. aureus USA300 JE2	1.5	1.55
	S. aureus Newman	1.5	1.55
	S. aureus RN4220	0.9	0.93
	S. aureus NCTC 8325	0.9	0.93
	S. epidermidis IVK7	6.0	6.22
	S. hominis 9VPs_KB1	4.6	4.77
	S. hominis 89VPS_B7	8.6	8.92
	S. capitis 50VAS_KB6	4.6	4.77
	S. warneri 1929	3.7	3.83
	S. warneri 1930	6.0	6.22
	M. luteus	7.4	7.67
	S. pyogenes	0.6	0.62
Vancomycin	*S. aureus* USA300 LAC	6.0	4.14
	S. aureus USA300 JE2	3.7	2.55
Daptomycin	*S. aureus* USA300 LAC	4.0	2.47
	S. aureus USA300 JE2	4.3	2.66
Lugdunin	*S. aureus* USA300 LAC	7.3	9.32
	S. aureus USA300 JE2	8.5	10.9

Assays were performed on TSA inoculated with the indicated bacterial strain. *S. aureus* JE2 is a plasmid-cured descendant of *S. aureus* USA300 LAC.

* IC was determined by an agar diffusion assay as described in the Materials and Methods section.

Supplementary Table 4: Mutations identified by DNA sequencing of *S. aureus* USA300 after experimental evolution

Mutations unique to *S. aureus* vs IVK83										Frequency	
Chrom	REF	ALT	STRAND	NT_POS	AA_POS	EFFECT	LOCUS_TAG	GENE	PRODUCT	*S. aureus* (vs IVK83)	*S. aureus* (vs IVK83 Δ*efiTP*)
1	G	A	+	1417/1482	473/493	A473T	NMEDEPAD_00746	*cls_1*	Cardiolipin synthase	0,2	0
1	T	A	-	188/975	63/324	K63I	NMEDEPAD_01074	*yqeN*	putative protein YqeN	0,2	0
1	G	A	+	1324/1485	442/494	G442K	NMEDEPAD_00995	*zwf*	Glucose-6-phosphate 1-dehydrogenase	0,8	0
1	C	T	+	718/807	240/268	Q240*	NMEDEPAD_00611		hypothetical protein	0,4	0
1	G	A	-	1480/1767	494/588	H494Y	NMEDEPAD_01115	*aspS*	Aspartate--tRNA ligase	0,8	0
1	C	T	+	485/1092	162/363	A162V	NMEDEPAD_00749	*desK*	Sensor histidine kinase DesK	0,4	0
1	CCGT	ACGG	+	284/1092	95/363	AV95DG	NMEDEPAD_00749	*desK*	Sensor histidine kinase DesK	0,6	0
2	G	T	+	455/933	152/310	R152L	NMEDEPAD_01976	*lacC_2*	Tagatose-6-phosphate kinase	0,8	0

Mutations common to *S. aureus* vs IVK83 and *S. aureus* vs IVK83 Δ*efiTP*										Frequency	
Chrom	REF	ALT	STRAND	NT_POS	AA_POS	EFFECT	LOCUS_TAG	GENE	PRODUCT	*S. aureus* (vs IVK83)	*S. aureus* (vs IVK83 Δ*efiTP*)
1	A	G	+	1135/1485	379/494	S379G	NMEDEPAD_00066	*yhdG_1*	putative amino acid permease YhdG	1	1
5	A	C	+	117/300	39/99	R39S	NMEDEPAD_02274	*yezG_1*	putative antitoxin YezG	1	1
10	C	T	+	274/483	92/160	P92S	NMEDEPAD_02627		hypothetical protein	0,2	1
9	AT	TATTC								1	1
9	A	ATTTAAAAATGG								1	1

Allele frequencies were determined with a score=1 if present in all 5 clones.

209 **Supplementary Table 5: Bacterial strains used in this study**

Bacterial species/strains	Origin/description	Source or reference
Bacillus cereus (group)	Various human nasal isolates	Peschel lab strain collection
Citrobacter freundii	Various human nasal isolates	Peschel lab strain collection
Citrobacter koseri	Various human nasal isolates	Peschel lab strain collection
Corynebacterium aurimucosum		
10VPs_Sm8	Human nasal isolate	[67]
Corynebacterium pseudodiphteriticum	Various human nasal isolates	Peschel lab strain collection; [66]
Cutibacterium acnes	Various human nasal isolates	[67]
Dermabacter hominis	Human nasal isolate	Peschel lab strain collection
Dolosigranulum pigrum		
9VAs_B4	Human nasal isolate	[67]
Enterococcus faecium	Various human isolates	Medical Microbiology, Tübingen
Escherichia coli		
DH5α	K-12 derivative	New England BioLabs
DC10B	Δ*dcm* in the DH10B background	[68]
Klebsiella oxytoca	Human nasal isolate	Peschel lab strain collection
Klebsiella pneumoniae	Various human nasal isolates	Peschel lab strain collection
Kocuria sp.	Various human nasal isolates	Peschel lab strain collection
Micrococcus luteus	Various human nasal isolates	Peschel lab strain collection
Moraxella catarrhalis		
44VAs_Sm4	Human nasal isolate	[67]
80VAs_B4	Human nasal isolate	[67]
Moraxella nonliquefaciens	Various human nasal isolates	Peschel lab strain collection
Raultella ornitholytica	Various human nasal isolates	Peschel lab strain collection
Staphylococcus aureus	Various human nasal isolates	[13]
USA300 LAC	Community-acquired (CA)-MRSA isolate	[69]

	Newman		[70]
	Newman Strep^R	Streptomycin-resistent *S. aureus* Newman	[25]
	USA300 JE2	CA-MRSA isolate	
		desK-A162V	this work
		desK-AV95DG	this work
	RN4220		[71]
	NCTC8325		Culture Collections Public Health England, Porton Down, UK
Staphylococcus capitis			
	10VAs_KB2	Human nasal isolate	[67]
	44UNs_B2	Human nasal isolate	[67]
Staphylococcus caprae			
	VA18305/14	Human clinical isolate	[72]
	BK3880/14	Human clinical isolate	[72]
Staphylococcus carnosus			
	TM300		
Staphylococcus epidermidis		Various human nasal isolates	This study
	IVK83	Human nasal isolate	[13]
	IVK83 Δ*efiTP*	Deletions of Δ*efiTP*	This study
	IVK83 Δ*efiTP* pRB474-83compl	Complementation of Δ*efiTP*	This study
Staphylococcus hominis			
	50MNs_Sa6	Human nasal isolate	[67]
	89VPs_B7	Human nasal isolate	[67]
	9VPs_KB1	Human nasal isolate	[67]
Staphylococcus lugdunensis			
	HKU09-01	Human skin infection isolate	[73]
	IVK28	Human nasal isolate	[13]
Staphylococcus pettenkofferi			
	210-70229632	Human clinical isolate	Peschel lab strain collection
Staphylococcus sciuri (now *Mammaliicoccus sciuri*)			
	50VAs_KB6	Human nasal isolate	[67]
Staphylococcus warneri			

VA16066/14	Human clinical isolate	[72]
BK6091/14	Human clinical isolate	[72]
Staphylococcus xylosus		
C2a		[74]
Streptococcus pyogenes		
BK2192		Peschel lab strain collection
**Fungi**		
Candida albicans		Medical Microbiology, Tübingen
Saccharomyces cerevisiae		

Supplementary Table 6: Plasmids used in this study

Plasmid	Description	Reference
pBASE6	Thermosensitive vector for allelic exchange	[62]
pRB474	*E. coli/S. aureus* shuttle vector; constitutively active expression vector	[64]
pIVK83	Plasmid containing the BGC for epifadin	This study
pTV1ts	Plasmid containing Tn917 (*ermR*)	[53]

Supplementary Table 7: Oligonucleotides used in this study

Oligonucleotides	Sequence (5'-3')	Purpose
83 KO Acc65I	AATGGTACCAAAATAATTTGCATTGTTT	Generating upstream recombinant region for *efiTP* deletion
83 KO EcoRI	TAGAATTCATATCAGCTTAGTTATATATTA	Generating upstream recombinant region for *efiTP* deletion
83 KO BssHI	AAGCGCGCATTCTTTCTTTCACATAATTTGC	Generating downstream recombinant region for *efiTP* deletion
83 KO SalI	TAATGTCGACTAAAATTTCCAATTTCGG	Generating downstream recombinant region for *efiTP* deletion
83 compl BamHI	AACTGGATCCAGGAAAAGAGGAAAACAATGC	Generating *efiTP* genes for complementation with pRB474
83 compl EcoRI	AGAAGAATTCGTATAAATATCGGACTTATTATGTGC	Generating *efitp* genes for complementation with pRB474
Tn917 up	CTGCAATAACCGTTACCTGTTTGTGCC	Screening for Tn917 insertion site
Ptn2 down	GGCCTTGAAACATTGGTTTAGTGGG	Screening for Tn917 insertion site

Supplementary references

67 Kaspar, U. *et al.* The culturome of the human nose habitats reveals individual bacterial fingerprint patterns. *Environ Microbiol* **18**, 2130-2142 (2016). https://doi.org:10.1111/1462-2920.12891

68 Monk, I. R., Shah, I. M., Xu, M., Tan, M. W. & Foster, T. J. Transforming the untransformable: application of direct transformation to manipulate genetically Staphylococcus aureus and Staphylococcus epidermidis. *mBio* **3** (2012). https://doi.org:10.1128/mBio.00277-11

69 Diep, B. A. & Otto, M. The role of virulence determinants in community-associated MRSA pathogenesis. *Trends Microbiol* **16**, 361-369 (2008). https://doi.org:10.1016/j.tim.2008.05.002

70 Duthie, E. S. & Lorenz, L. L. Staphylococcal coagulase; mode of action and antigenicity. *J Gen Microbiol* **6**, 95-107 (1952). https://doi.org:10.1099/00221287-6-1-2-95

71 Kreiswirth, B. N. *et al.* The toxic shock syndrome exotoxin structural gene is not detectably transmitted by a prophage. *Nature* **305**, 709-712 (1983). https://doi.org:10.1038/305709a0

72 Winstel, V., Kuhner, P., Krismer, B., Peschel, A. & Rohde, H. Transfer of plasmid DNA to clinical coagulase-negative staphylococcal pathogens by using a unique bacteriophage. *Appl Environ Microbiol* **81**, 2481-2488 (2015). https://doi.org:10.1128/AEM.04190-14

73 Tse, H. *et al.* Complete genome sequence of Staphylococcus lugdunensis strain HKU09-01. *J Bacteriol* **192**, 1471-1472 (2010). https://doi.org:10.1128/JB.01627-09

74 Sizemore, C., Wieland, B., Gotz, F. & Hillen, W. Regulation of Staphylococcus xylosus xylose utilization genes at the molecular level. *J Bacteriol* **174**, 3042-3048 (1992). https://doi.org:10.1128/jb.174.9.3042-3048.1992

75 Pang, X. *et al.* New Tetramic Acid Derivatives From the Deep-Sea-Derived Fungus Penicillium sp. SCSIO06868 With SARS-CoV-2 M(pro) Inhibitory Activity Evaluation. *Front Microbiol* **12**, 730807 (2021). https://doi.org:10.3389/fmicb.2021.730807

76 Cheng, J. T. *et al.* Discovery of a Potential Liver Fibrosis Inhibitor from a Mushroom Endophytic Fungus by Genome Mining of a Silent Biosynthetic Gene Cluster. *J Agric Food Chem* **69**, 11303-11310 (2021). https://doi.org:10.1021/acs.jafc.1c03639

77 Drescher, C., Keller, M., Potterat, O., Hamburger, M. & Brückner, R. Structure-Elucidating Total Synthesis of the (Polyenoyl)tetramic Acid Militarinone C§. *Org Lett* **22**, 2559-2563 (2020). https://doi.org:10.1021/acs.orglett.0c00431

78 Drescher, C. & Bruckner, R. Structure-Proving Syntheses of the Polyenoyltetramic Acids Pyranonigrin J and I. *Eur J Org Chem* **2022** (2022). https://doi.org:ARTN e202101053 10.1002/ejoc.202101053

79 Reeves, C. D. *et al.* Alteration of the substrate specificity of a modular polyketide synthase acyltransferase domain through site-specific mutations. *Biochemistry* **40**, 15464-15470 (2001). https://doi.org:10.1021/bi015864r

80 Chang, Y., Wang, Y.-H. & Hu, C.-Q. Simultaneous determination of purity and potency of amphotericin B by HPLC. *The Journal of Antibiotics* **64**, 735-739 (2011). https://doi.org:10.1038/ja.2011.83

81 Ramachandran, G. *et al.* Discovery of a new family of relaxases in Firmicutes bacteria. *PLoS Genet* **13**, e1006586 (2017). https://doi.org:10.1371/journal.pgen.1006586
