# Supplementary material for: Commensal production of a broad-spectrum and short-lived antimicrobial peptide polyene eliminates nasal Staphylococcus aureus
Source: Nat Microbiol. 2023 Dec 18;9(1):200–13. doi: 10.1038/s41564-023-01544-2 (PMC11310079; doi:10.1038/s41564-023-01544-2)
Supplement: Supplementary file 2 — Reporting Summary [file 41564_2023_1544_MOESM2_ESM.pdf]

nature portfolio

Corresponding author(s): Dr. Bernhard Krismer
Last updated by author(s): Oct 26, 2023

Reporting Summary

Nature Portfolio wishes to improve the reproducibility of the work that we publish. This form provides structure for consistency and transparency in reporting. For further information on Nature Portfolio policies, see our Editorial Policies and the Editorial Policy Checklist.

Statistics

For all statistical analyses, confirm that the following items are present in the figure legend, table legend, main text, or Methods section.

n/a	Confirmed	
☐	☒	The exact sample size (*n*) for each experimental group/condition, given as a discrete number and unit of measurement
☐	☒	A statement on whether measurements were taken from distinct samples or whether the same sample was measured repeatedly
☐	☒	The statistical test(s) used AND whether they are one- or two-sided<br>*Only common tests should be described solely by name; describe more complex techniques in the Methods section.*
☒	☐	A description of all covariates tested
☒	☐	A description of any assumptions or corrections, such as tests of normality and adjustment for multiple comparisons
☐	☒	A full description of the statistical parameters including central tendency (e.g. means) or other basic estimates (e.g. regression coefficient) AND variation (e.g. standard deviation) or associated estimates of uncertainty (e.g. confidence intervals)
☐	☒	For null hypothesis testing, the test statistic (e.g. *F*, *t*, *r*) with confidence intervals, effect sizes, degrees of freedom and *P* value noted<br>*Give P values as exact values whenever suitable.*
☒	☐	For Bayesian analysis, information on the choice of priors and Markov chain Monte Carlo settings
☒	☐	For hierarchical and complex designs, identification of the appropriate level for tests and full reporting of outcomes
☒	☐	Estimates of effect sizes (e.g. Cohen's *d*, Pearson's *r*), indicating how they were calculated

Our web collection on statistics for biologists contains articles on many of the points above.

Software and code

Policy information about availability of computer code

Data collection	No commercial, open source or custom code was used to collect data for this study.
Data analysis	antiSMASH 5.0 (bacterial version) was used to analyse the epifadin biosynthetic gene cluster; Whole-genome sequence of S. epidermidis IVK83 was determined by Illumina short-read and PacBio long-read sequencing; Illumina reads were de-novo assembled by velvet (version 1.2.10); Alignment of the two de-novo assemblies with MAUVE (version 2.4.0) and subsequent manual curation allowed us to generate the final genome, which was confirmed by mapping the Illumina reads to the final assembly. The circular chromosome and the plasmid were annotated using the NCBI Prokaryotic Genome Annotation Pipeline (version 5.3) and deposited at NCBI. DNA analysis was performed with DNASTAR Lasergene (version 15); ImageJ software (version 1.8.0_112) was used to measure zones of inhibition for stability analysis of epifadin. GraphpadPrism 8 was used to generate figures and for statistical analyses. For WGS of isolates from experimental evolution, Trimmomatic (v0.39) was used to trim adapters and low-quality bases and read qualities were assessed using FastQC v0.11.7 (https://www.bioinformatics.babraham.ac.uk/projects/fastqc/) and MultiQC v1.0. Genome sequences were assembled de novo and annotated using Unicycler v 0.4.7 with default parameters, using SPAdes v 3.15.4, and Prokka v 1.14.6.

For manuscripts utilizing custom algorithms or software that are central to the research but not yet described in published literature, software must be made available to editors and reviewers. We strongly encourage code deposition in a community repository (e.g. GitHub). See the Nature Portfolio guidelines for submitting code & software for further information.

Data

Policy information about availability of data

All manuscripts must include a data availability statement. This statement should provide the following information, where applicable:

- Accession codes, unique identifiers, or web links for publicly available datasets
- A description of any restrictions on data availability
- For clinical datasets or third party data, please ensure that the statement adheres to our policy

All data supporting the findings of this study are available within the paper, its Extended data or Supplementary information. WGS data obtained for S. epidermidis IVK83 were deposited in the NCBI Sequence Read Archive (genome available under accession number CP088002, plasmid pIVK83 under CP088003). Sequence of strain S. epidermidis B155 (Liverpool, UK) was deposited as BioSample SAMEA12384066 (BioProject PRJEB50307). Representative microscopy images are included in the extended data figures and the supplementary videos, which were deposited at Figshare (doi.org/10.6084/m9.figshare.24125589). NMR data were deposited at nmrXive and are available under the project identifier NMRXIV:P18 (10.57992/nmrxiv.p18; https://nmrxiv.org/P18). Source data for experiments is provided.

Field-specific reporting

Please select the one below that is the best fit for your research. If you are not sure, read the appropriate sections before making your selection.

☒ Life sciences ☐ Behavioural & social sciences ☐ Ecological, evolutionary & environmental sciences

For a reference copy of the document with all sections, see nature.com/documents/nr-reporting-summary-flat.pdf

Life sciences study design

All studies must disclose on these points even when the disclosure is negative.

Sample size	Epifadin instability testing, in vitro competition assays, minimal bactericidal concentration assay and cytotoxicity assay were chosen to have a sample size of 3 independent replicates, as this is the minimum number for statistical testing. The sample size of cotton rats used for S. epidermidis IVK83 wildtype and S. epidermidis ΔefiTP nasal colonization was chosen to be 11 animals (5 and 6 per group, respectively), as this number is necessary to determine the median colonization capability of bacterial strains in cotton rat noses. The sample size of cotton rats used for S. epidermidis IVK83 wildtype + S. aureus Newman and S. epidermidis ΔefiTP + S. aureus Newman nasal co-colonization was chosen to be 17 animals (9 and 8 per group, respectively), as this number was sufficient to observe that S. epidermidis IVK83 wildtype is capable to reduce S. aureus nasal colonization in contrast to S. epidermidis ΔefiTP. Here, we kept sample sizes identical or similar to previously published experiments (https://pubmed.ncbi.nlm.nih.gov/27466123)
Data exclusions	No data was excluded for any of the experiments.
Replication	Replication of epifadin instability testing, in vitro competition assays, minimal bactericidal concentration assay and cytotoxicity assay was performed with n=3 biological replicates and was successful each time. Epifadin inhibitory concentration determination was performed once, but in parallel with all strains, due to the limited availability of purified epifadin. Membrane depolarisation assays were performed with n=2 biological replicates with n=2 technical replicates, each.
Randomization	Randomization was only partially conducted since female cotton rats are usually kept in groups of two or three animals per cage. All animals from one cage had to be colonised with the same bacterial strain, or strain-combination. All male animals were kept alone in individual cages and were randomly colonised.
Blinding	Animal experiments could be performed only by two experimenters who had to work together, for which reason blinding in the animal facility was not possible. Sample plating was performed by another person who was not involved in the colonisation. Colony counting was performed by two methods, a semiautomatic camera-based, which resulted in absolute colony numbers, and a manual counting which distinguished between S. aureus and S. epidermidis. This differentiation between colony morphologies was totally objective.

Reporting for specific materials, systems and methods

We require information from authors about some types of materials, experimental systems and methods used in many studies. Here, indicate whether each material, system or method listed is relevant to your study. If you are not sure if a list item applies to your research, read the appropriate section before selecting a response.

Materials & experimental systems

n/a	Involved in the study	
☒	☐	Antibodies
☐	☒	Eukaryotic cell lines
☒	☐	Palaeontology and archaeology
☐	☒	Animals and other organisms
☒	☐	Human research participants
☒	☐	Clinical data
☒	☐	Dual use research of concern

Methods

n/a	Involved in the study	
☒	☐	ChIP-seq
☒	☐	Flow cytometry
☒	☐	MRI-based neuroimaging

Eukaryotic cell lines

Policy information about cell lines

Cell line source(s)	Human cervical carcinoma HeLa cell line (ATCC)
Authentication	Cell line was initially purchased by ATCC. The cell line was recently analysed by the DSMZ via DNA profiling using 17 different and highly polymorphic short tandem repeat (STR) loci. This confirmed the identity as HeLa without any doubt.
Mycoplasma contamination	Cell lines were not tested for mycoplasma contamination
Commonly misidentified lines (See ICLAC register)	In this study no commonly misidentified cell lines were used.

Animals and other organisms

Policy information about studies involving animals; ARRIVE guidelines recommended for reporting animal research

Laboratory animals	In this study, cotton rats (Sigmodon hispidus) of both sexes, 8-12 weeks old, were used.
Wild animals	No wild animals were used in this study.
Field-collected samples	The study did not involve samples collected from the field.
Ethics oversight	All animal experiments were conducted in strict accordance with the German regulations of the Gesellschaft für Versuchstierkunde/ Society for Laboratory Animal Science (GV-SOLAS) and the European Health Law of the Federation of Laboratory Animal Science Associations (FELASA) in accordance with German laws after approval by the local authorities (IMIT 1/15, Regierungspräsidium Tübingen)

Note that full information on the approval of the study protocol must also be provided in the manuscript.
